# Efficient Data Transfer and Multi-Bit Multiplier Design in Processing in Memory

**DOI:** 10.3390/mi15060770

**Published:** 2024-06-09

**Authors:** Jingru Sun, Zerui Li, Meiqi Jiang, Yichuang Sun

**Affiliations:** 1 Chongqing Research Institute, Hunan University, Chongqing 401120, China; 2 College of Computer Science and Electronic Engineering, Hunan University, Changsha 410082, China; lzr98111@hnu.edu.cn (Z.L.); meiqij@hnu.edu.cn (M.J.); 3 School of Engineering and Computer Science, University of Hertfordshire, Hatfield AL10 9AB, UK; y.sun@herts.ac.uk

**Keywords:** memristor, multiplier, adder, crossbar array, MPU, PiM

## Abstract

Processing in Memory based on memristors is considered the most effective solution to overcome the Von Neumann bottleneck issue and has become a hot research topic. The execution efficiency of logical computation and in-memory data transmission is crucial for Processing in Memory. This paper presents a design scheme for data transmission and multi-bit multipliers within MAT (a data storage set in MPU) based on the memristive alternating crossbar array structure. Firstly, to improve the data transfer efficiency, we reserve the edge row and column of the array as assistant cells for OR AND (OA) and AND data transmission logic operations to reduce the data transfer steps. Furthermore, we convert the multipliers into multi-bit addition operations via Multiple Input Multiple Output (MIMO) logical operations, which effectively improves the execution efficiency of multipliers. PSpice simulation shows that the proposed data transmission and multi-bit multiplier solution has lower latency and power consumption and higher efficiency and flexibility.

## 1. Introduction

Processing-in-memory (PiM) architecture based on new devices has become a critical solution to address the Von Neumann bottleneck and Moore’s law limit problems [1]. Among them, memristor has become the most promising technology for solving this problem due to its non-volatile nature, small size, low power consumption, and easy integration with CMOS technology [2].

Chua proposed the memristor concept in 1971 [3]. In 2008, HP manufactured the first memristor device [4], which has since attracted extensive interest from researchers. Research on memristors involves the study of devices [5], mathematical models [6], and circuit designs [7,8]. These works form the basis for the practical implementation of memristors. Currently, memristors have been utilized in a variety of fields [9,10]. For example, memristors are particularly well-suited for the design of synaptic circuits in neural morphological networks because of their nonlinear and resistance variability characteristics [11,12,13]. Yuan et al. proposed a highly efficient neuromorphic physiological signal processing system based on memristors [14]. Lin et al. used three-dimensional memristor circuits to create complex neural networks [15]. The nonlinear characteristics of memristors bring more possibilities to the design of chaotic circuits [16]. Bao et al. proposed a memristor-based neuron model [17], Zhang et al. generated a multitude of diverse hidden attractors through the coupling of memristors [18], and Ma et al. analyzed the synchronization in scale-free neural networks under electromagnetic radiation by memristor [19].

Memristors are a preferred choice for studying new types of non-volatile memory due to their non-volatile characteristics [20]. In 2014, Zangeneh et al. proposed a memristor memory with a crossbar array called 1T1M, based on which memristor-based multivalued memories are designed [21,22]. Additionally, Sun et al. proposed a 3D memristive multivalue memory [23]. Currently, the crossbar array is the main structure of memristive memory, and different memory cells, including 1T1M (one transistor, one memristor) [24], 1T2M [25], 2M1M [26], 2M2M [23], and so on, are proposed to increase the storage density.

**Figure 1:**
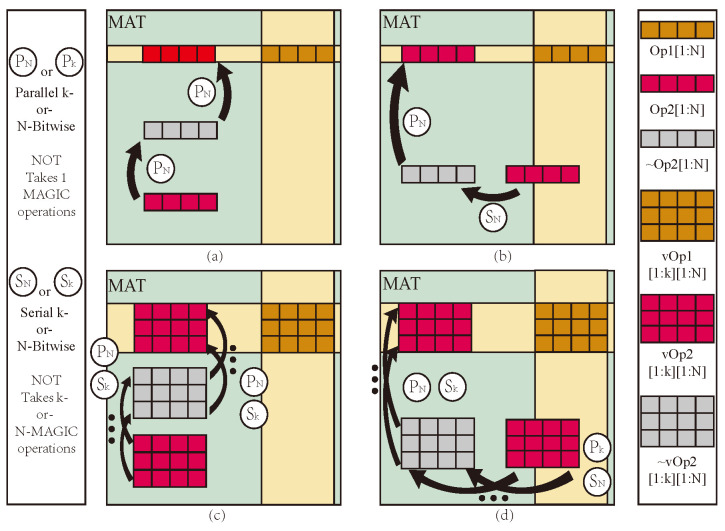
MAT Structure and data transfer: (**a**) Op1 shares data lines with Op2, (**b**) Op1 shares part data lines with Op2, (**c**) vOp1 shares data lines with vOp2, (**d**) vOp1 share part data lines with vOp2.

Research on PiM based on memristors has gained significant attention [27]. Among the various approaches explored, the 1T1M crossbar array stands out due to its simple structure, high stability, and ease of implementation for logic operations [28]. This approach has provided a reliable foundation for developing PiM logic operation research. In 2016, the neural network accelerator prime based on memristor random access memory (ReRAM) was designed and implemented, and the memory computing integrated architecture based on memristor was determined [29]. Subsequently, Wu et al. proposed a 128×128 memristor-based analog synapse crossbar array to realize face classification [30]. Furthermore, Hur et al. proposed the concept of a memory processing cell (MPU) with memristor [31]. In 2018, Talati et al. further discussed data transfer and parallelism based on a memristor MPU [32], as shown in Figure 1. However, this work uses memristor-aided logic (MAGIC) NOT as the basic logical operation, and data transmission to the destination position requires at least two steps of NOT operation, which seriously affects the operational efficiency of PiM.

The logic operation based on the memristor is the basis of realizing memristor PiM technology. Regarding the effective memristor logic design, the design of Sun et al. [33] has realized the most efficient design of all logic gates, with the least number of devices and the least operation steps. The memristor logic operation can be divided into two categories. One is the logic operation structure based on a hybrid memristor–CMOS, such as the memristor adder circuit [34] and the memristor multiplier circuit [35]. These circuits use the nonvolatile nature of memristors to achieve faster particular logic operations but lack versatility. The second type is the logic operation based on memristor crossbar array, which has become the prominent architecture of PiM due to its good versatility. Realizing efficient logic operation in an array structure is the core problem of PiM design.

Material implication (IMPLY) [36] is built based on a crossbar array structure. It is simple, reliable, and can implement complete Boolean logic operations, but its efficiency is relatively low. Kvatinsky et al. proposed MAGIC logic [37], which separates operand and result and can implement logic gates such as IMP, XNOR, NAND, and OR. Jiang et al. proposed an MIMO logic gate based on IMPLY [38], which can derive multiple new efficient logic operation methods and complete complex logic with fewer steps and memristors.

The computing system includes many fundamental logic blocks. The full adder (FA) is one of the most frequently used blocks. FA implementations include serial and parallel full adders. The serial FA requires fewer memristors but has multiple operating steps and low efficiency [39]. On the other hand, the parallel FA requires fewer execution steps but involves more memristors in the calculation [40]. A semiparallel FA has been proposed as a solution to achieve balance [41]. In 2023, Jiang et al. proposed alternating crossbar array parallel FA based on MIMO logic, and alternating crossbar array achieves faster execution speed and fewer memristors [38].

Multiplier is another complex logic operation module used in convolution, digital filtering, and fast Fourier transform (FFT) applications [42]. It has significant complex logical operations and generates long-chain combination blocks with cascaded carry addition. Improving basic computing efficiency and reducing the carry step are the keys to achieving a high-efficiency multiplier.

Multipliers based on memristor crossbar array structures can be divided into matrix and exact multi-bit multipliers. Matrix operation is designed for matrix computing needs, such as convolution and image processing. This type of multiplier has high execution efficiency and good universality, but the calculation results are not accurate enough due to the analog signal operation mode [43]. Unlike matrix multiplication, multi-bit multiplication is mainly aimed at computer logic operations and must provide accurate calculation results. Therefore, research on multi-bit multipliers starts with cell logic gates such as IMPLY and MAGIC and gradually completes the entire operation. For instance, Saeed et al. developed a binary multiplier that utilized the resistive characteristic of a memristor [44]. Guckert et al. proposed a Dadda memristor multiplier; each cell contains two memristors and can achieve IMPLY operation in one cell [45]. Furthermore, Mehri et al. proposed a special memristor–CMOS multiplier [46]. However, the structure requires more CMOS switches and increased chip area. Yu et al. proposed a current-mode multi-memristor crossbar cell multiplier that effectively reduces power consumption. However, the calculation process is intricate [47]. Radakovits et al. proposed a multiplier using semi-serial FA [48], which has the least memristors and switches, but the execute steps are still high. Constructing a highly efficient multiplier with fewer devices and lower power consumption remains challenging.

This paper focuses on PiM MAT data transfer and logic operation efficiency and proposes a high-efficiency data transfer method and multiplier logic operation structure based on a memristor alternating crossbar array. The main works are as follows:

- Improved data transfer efficiency in PiM MPU by reserving a row and column at the edge of the array for logic assistant based on AND and OA logic.
- A multi-bit multiplier with fewer execution steps and devices and lower latency and power consumption is designed based on alternating crossbar array architecture and MIMO logic operations.

The paper is structured as follows: In Section 2, we introduce the memristor model, MIMO memristive logic, PiM structure, and the multiplier’s working mechanism. Section 3 describes the proposed data transfer within the MAT and the multiplier design method. In Section 4, we showcase the correctness of our design through PSpice simulation. In addition, Section 5 presents a comparison between different multipliers. Finally, in Section 6, we conclude the paper.

## 2. Materials and Methods

### 2.1. Data Transfer in MPU

**Figure 2:**
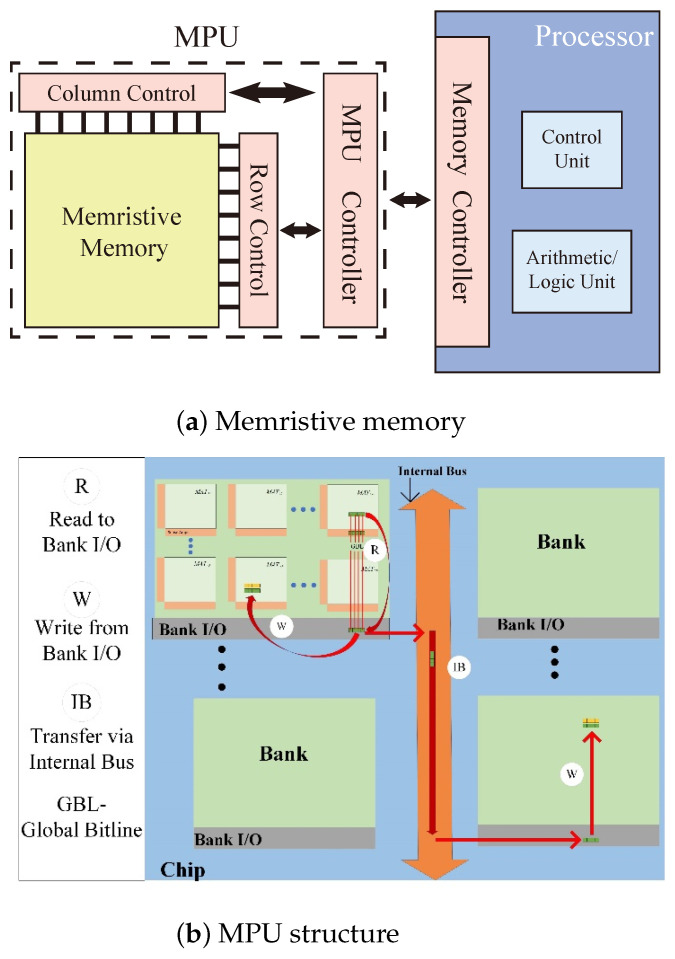
PiM Structure.

The PiM architecture shown in Figure 2a includes a processor and a memristive memory processing cell (mMPU) [31,32]. The mMPU contains an mMPU controller and memristive memory. The mMPU controller is responsible for receiving instructions from the processor and generating the control signals for the mMPU to perform reads, writes, and logical operations. Memristive memory does not physically separate processing and memory spaces and directly employs the memory cells for computation; it does not need to transfer data from the memory array.

However, one constraint of processing operands in the mMPU is that their physical addresses should share the same word lines/bit lines (WLs/BLs) since they serve as circuit connections among the inputs and outputs. If two operands that need to be processed are present in different WLs/BLs, they first need to be copied to addresses that share WLs/BLs with other operands.

Data in the mMPU is organized hierarchically. A two-dimensional array of memristive memory cells forms an mMPU-MAT. Collections of several such MATs form a Bank, as shown in Figure 2b, and multiple Banks are gathered to form a chip with a common internal bus shared among Banks. In the mMPU, data must be moved within and across different MATs, especially in MAT, depending on the operand locality and their alignment inside a MAT.

Based on the physical addresses of input and output operands, data transfer is categorized as follows:

- Intra-MAT refers to a situation where the operands must be transferred within a MAT.
- Intra-Bank refers to a situation where operands are transferred between MATs where the operands must be transferred from one MAT to another within the same Bank.
- Inter-Bank refers to the operands between Banks that must be transferred from one Bank to another within the same chip.

Intra-MAT is the foundation of logical operations and storage, and its transmission efficiency is crucial to the execution efficiency of mMPU. When performing logical operations in a MAT, two operands must be aligned in the same row. Regarding two operands, suppose Op2 either does not share data lines with Op1 (Figure 1a) or only partially shares BLs with Op1 (Figure 1b); Op2 must be moved to align with Op1 within the MAT at a desired location. In the first case, two parallel MAGIC NOT operations can align Op2 with Op1. In contrast, in the latter case, Op2 first needs to be moved to a temporary memory location using a serial sequence of MAGIC NOT operations because of the inherent limitation of gates not being able to perform multiple operations in the same WL simultaneously. For vector operations with vectors vOp1 and vOp2, having N-elements each and assuming that all the elements are present in a vector form, the cost of intra-MAT data transfer is even higher since MAGIC NOT operations have to be performed element-wise (Figure 1d). In total, the latency cost of intra-MAT data alignment for vector operations is

(1) $$C^{\mathrm{intra}-\mathrm{MAT}\;}=\left\{\begin{array}{c} \min\left\{2k,k+N\right\}\cdot T_{logic},\;\mathrm{no}\;\mathrm{overlap}\;\\ \left(k+N\right)\cdot T_{logic},\;\;\mathrm{partial}\;\mathrm{overlap}\; \end{array}.\right.$$

*N* is the number of elements in each vector operation, and *K* is the vector number. $\;T_{logic}$ is the latency of MAGIC operation.

### 2.2. Memristor Model

This paper uses the VTEAM memristor model, which is generic, simple, flexible, and adaptable to different memristor devices and has been widely used in the design of memristor circuits. The VTEAM model is represented as follows:

(2) $$\frac{dw(t)}{dt}=\left\{\begin{array}{cc} \;k_{off}*{\left(\frac{v(t)}{v_{off}}-1\right)}^{\alpha_{off}}*f\left(w\right), & v<v_{off}<0\\ \;0\\ \;k_{on}*{\left(\frac{v(t)}{v_{on}}-1\right)}^{\alpha_{on}}*f\left(w\right), & 0<v_{on}<v \end{array}\right.,$$

(3) $$i\left(t\right)={\left[R_{ON}+\frac{R_{OFF}-R_{ON}}{w_{off}-w_{on}}*\left(w-w_{on}\right)\right]}^{-1}*v\left(t\right),$$

where *w* denotes the internal state variable, $w\in [w_{on},w_{off}]$, v(t) and i(t) denote the voltage and current flowing through the memristor, respectively, and f(w) is expressed as a speed-adaptive function. Ron and Roff denote the resistance of the memristor in the low-resistance state (LRS) and high-resistance state (HRS), respectively. Typically, LRS is considered as logic 1 (off) and HRS is considered as logic 0 (on).

**Figure 3:**
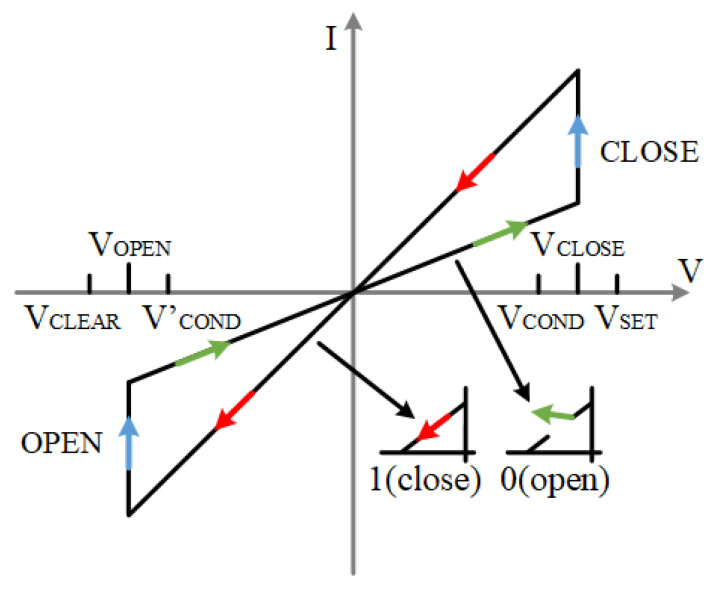
Memristor I–V characteristic curve under ideal model, red color arrows are the LRS state, green arrows are the HRS state, blue arrows are the changing state.

The I–V characteristic curve of the ideal memristor model is shown in Figure 3, where $V_{CLEAR}$ <$V{’}_{COND}$ <$V_{COND}$ <$V_{SET}$. These voltages have a certain range of values. According to Formulas (5)–(7) and (9)–(11). The voltage range of $V_{SET}$ is between [1.05, 1.38] and that of $V_{COND}$ is between [0.74, 0.96]. Similarly, the voltage range of $V{’}_{COND}$ is [−0.96, −0.74], and the voltage range of $V_{CLEAR}$ is [−1.38, −1.05]. The value range of $R_{g}$ is [328, 2000] and [277, 1518]. These ranges make the logic operation in our later experiments normal, and all kinds of devices are in a safe range.

When the applied voltage is greater than the positive threshold voltage, $V_{CLOSE}$, of the memristor, the memristor switches from state 0 to state 1; when the applied voltage is less than the negative threshold voltage, $V_{ON}$, of the memristor, the memristor switches from state 1 to state 0. This model is used for the memristors in the full adder as well as the multiplier in the rest of this paper.

### 2.3. Basic Logic Operation

The execution efficiency of basic logic operations plays a decisive role in the logic calculation speed of storage and computing integrated cells. This section studies the multi-input–multi-output universal basic logic design scheme with higher execution efficiency.

#### 2.3.1. Multi-Input Implicative Logic Circuit Design

IMPLY logic is inefficient, mainly because it only has two operands in the operation process. It needs more than two operands and more step operations when performing complex operations. We notice that IMPLY logic determines the input and output by applying different voltages to the input and output memristor. Moreover, IMPLY and AND logic have the same circuit structure, and different logic operations are realized by applying different voltages to the two input memristors.

**Figure 4:**
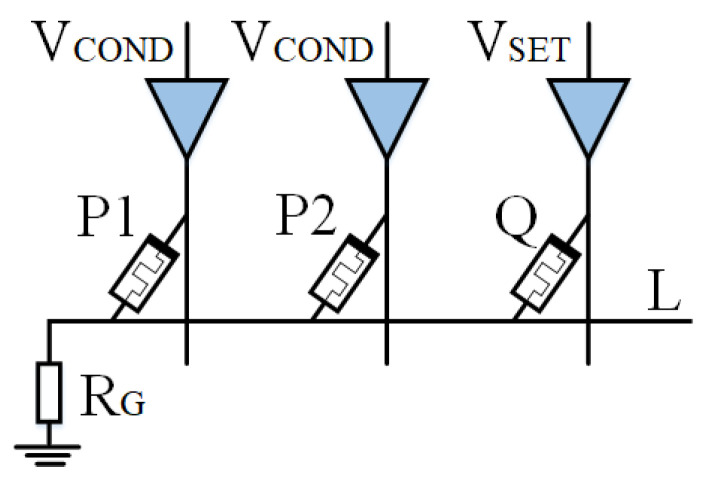
ONO logic circuit.

**Figure 5:**
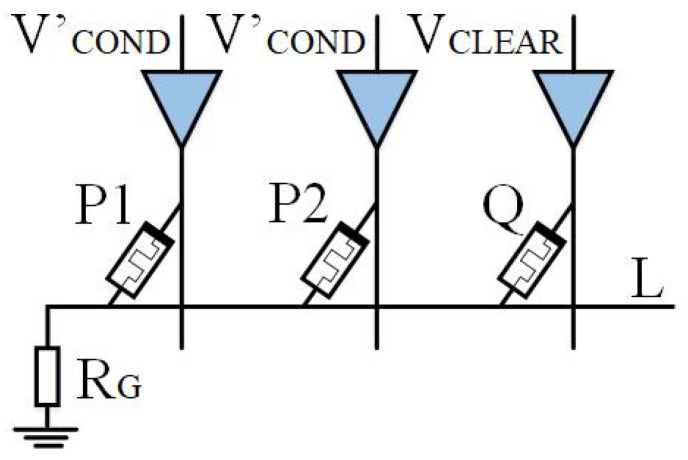
OA logic circuit.

Based on IMPLY, Jiang [22] and Huang et al. [49] then proposed a multi-input model. As shown in Figure 4 and Figure 5, a memristor P2 is added to the implication logic. Three inputs can be obtained according to the different input voltages, and various logic operations are derived. Here, we take two logics, OR NOT OR (ONO) logic and OR AND (OA) logic, as examples.

As shown in Figure 4, when $V_{COND}$ is applied to memristors P1 and P2, $V_{SET}$ is applied to memristor Q, P1 and P2 are input memristors, and Q is an output memristor. Logic $q=\bar{p_{1}+p_{2}}+q$ can be realized, which we call ONO (or nor or) logic operation, and its truth table is shown in Table 1.

The total parallel resistance of all input memristors is defined as resistance $R_{i}$, with high impedance state $R_{OFF}$ corresponding to logic 0 and low-resistance state $R_{ON}$ corresponding to logic 1, $R_{OFF}\gg R_{G}$. According to different situations, the analysis is as follows:

- Case 1: When memristors P1 and P2 are both in high impedance state (p1=p2=0), $R_{i}$ is equal to $\frac{R_{OFF}}{2}$. Because $R_{OFF}\gg R_{G}$, the voltage on $R_{G}$ is almost zero. Therefore, the voltage drop across the memristor Q is $V_{Q}$, wherein $V_{Q}\approx V_{SET}>V_{CLOSE}$. Therefore, the memristor Q is switched to a low-resistance state.
- Cases 2, 3, and 4: When one or two input memristors are in a low-resistance state, the resistance $R_{i}$ is less than the resistance $R_{ON}$. Because $R_{OFF}\gg R_{G}$, the voltage on $R_{G}$ is $V_{Q}$. The voltage drop across memristor Q is $V_{Q}\approx V_{SET}-V_{COND}<V_{CLOSE}$. The Q resistance state of memristor remains unchanged.

If the number of input memristors is extended to n, the logic becomes as follows. When the resistance state of all input memristors is 0, the parallel resistance is defined as logic 0, and the rest is defined as logic 1. We can obtain the resistance corresponding to the logic value.

(4) $$R_{i}=\left\{\begin{array}{cc} \frac{R_{OFF}}{n}, & \;\mathrm{logic}\;0\\ \left[\frac{R_{ON}}{n},\frac{R_{OFF}R_{ON}}{R_{OFF}+R_{ON}\left(n-1\right)}\right], & \;operandnamelogic1 \end{array}.\right.$$

Similar to the calculation method of implication logic, $R_{G}$ meets the following conditions:

(5) $$R_{G}<\frac{R_{OFF}\left(V_{SET}-V_{CLOSE}\right)}{\left(n+1\right)V_{CLOSE}-n\left(V_{SET}-V_{COND}\right)},$$

(6) $$R_{G}\geq \frac{R_{OFF}R_{ON}\left(V_{SET}-V_{CLOSE}\right)}{\left(R_{OFF}+nR_{ON}\right)V_{CLOSE}-\left[R_{OFF}+\left(n-1\right)R_{ON}\right]\left(V_{SET}-V_{COND}\right)}.$$

Input voltage meets the following conditions:

(7) $$V_{SET}-V_{COND}<\left(R_{OFF}+nR_{ON}\right)V_{CLOSE}.$$

Figure 5 represents the OA (or AND) logic. By applying $V_{COND}^{\prime }$ to input memristors P1 and P2, and $V_{CLEAR}$ to output memristor Q, the logic $q=(p_{1}+p_{2})\cdot q$ can be obtained. If there are n inputs, the logic becomes $q=(p_{1}+p_{2}\cdots +p_{n})\cdot q$. ONO and OA logic operations use the same circuit but different excitation voltages. The OA truth table can be found in Table 2.

Multi-input logic is a process of completing logical calculations for multiple input signals in one step by expanding the input memristor. Different logical operations can be flexibly realized by controlling the voltage applied to different memristors. The original input data can be retained by specifying the output Q memristor. Multi-input general logic models reduce the execution steps and have high computational efficiency and data reusability. They can be used to design complex logic operations that will effectively shorten computational time and reduce the number of memristors.

#### 2.3.2. Multi-Output Implicative Logic Circuit Design

The memristor Q is regarded as the output memristor in the basic logic operation circuit mentioned in the previous section. Expanding the output memristor in the design idea from the previous section can create a multi-output that can be used for various logical operations.

**Figure 6:**
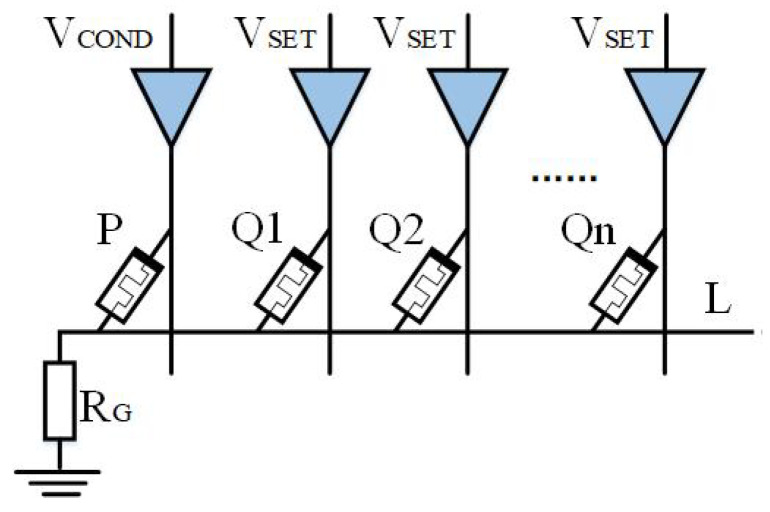
Multi-output logic circuit.

As shown in Figure 6, The output memristor with the logic circuit is extended to N, and the applied voltage of the output memristor is $V_{SET}$. When the circuit works, the initial logic values of $Q_{1}$,$Q_{2}$...,$Q_{n}$ should be guaranteed to be the same. IMPLY logic results are stored in n memristors, which means that $q_{1}=q_{2}=\cdots =q_{n}=\bar{p}+q$. The extended output of AND logic is $q_{1}=q_{2}=\cdots =q_{n}=p\cdot q$.

The parallel resistance of the output memristor is defined as

(8) $$R_{i}=\left\{\begin{array}{cc} \frac{R_{OFF}}{n}, & \;\mathrm{logic}\;0\\ \frac{R_{ON}}{n}, & \;operandnamelogic1 \end{array}.\right.$$

Similar to the calculation method of implication logic, we can obtain the constraint conditions of the extended output of implication logic.

(9) $$R_{G}<\frac{R_{OFF}\left(V_{SET}-V_{CLOSE}\right)}{\left(n+1\right)V_{CLOSE}-\left(V_{SET}-V_{COND}\right)},$$

(10) $$R_{G}\geq \frac{R_{ON}\left(V_{SET}-V_{CLOSE}\right)}{V_{CLOSE}\left(1+nR_{ON}/R_{OFF}\right)-\left(V_{SET}-V_{COND}\right)}.$$

Input voltage meets the following conditions:

(11) $$V_{SET}-V_{COND}<\left(1+nR_{ON}/R_{OFF}\right)V_{CLOSE}.$$

By satisfying inequalities (9)–(11), the extended output of implication logic can be realized. The extended output of AND logic is similar to implication logic.

Multi-output AND logic has multiple outputs, which means the operation results are stored in multiple memristors. When the output data is involved in multiple operations, data loss and data transmission time can be reduced. In addition, multi-input and multi-output logic operations can be realized in the same structure if the specific conditions of $R_{i}$ are met.

### 2.4. Alternating Crossbar Array

**Figure 7:**
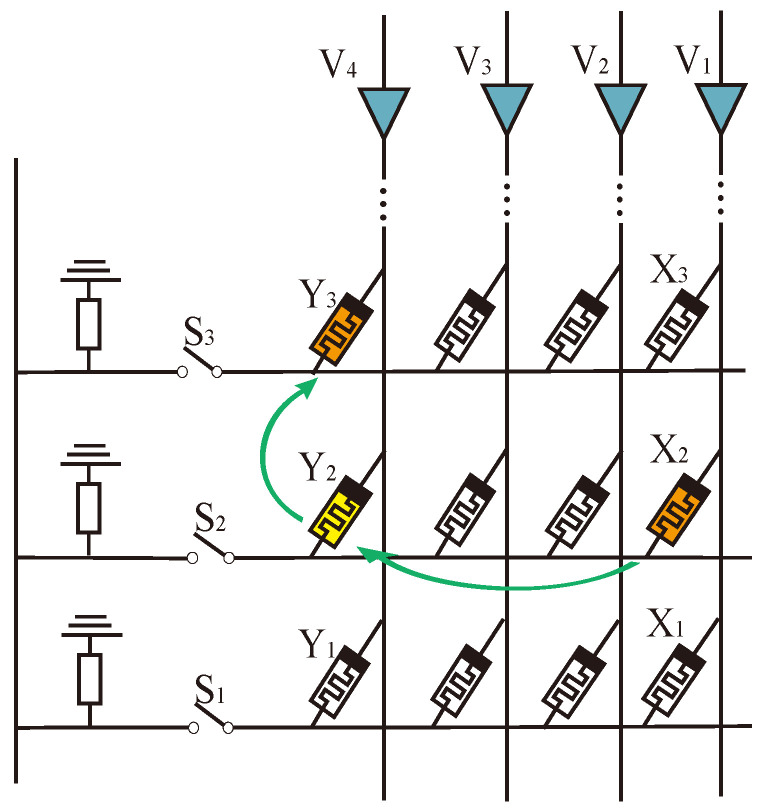
IMPLY operation between different rows in traditional crossbar array.

Figure 7 shows the structure of a traditional crossbar array. In this structure, when the logical operation involves different rows of memristors, it usually needs multiple steps. For example, it needs two steps to calculate $X_{2}$ IMPLY $Y_{3}$. First, copy $X_{2}$ to $Y_{2}$ by horizontal AND operation, and then IMPLY $Y_{2}$ to $Y_{3}$ by vertical IMPLY operation.

**Figure 8:**
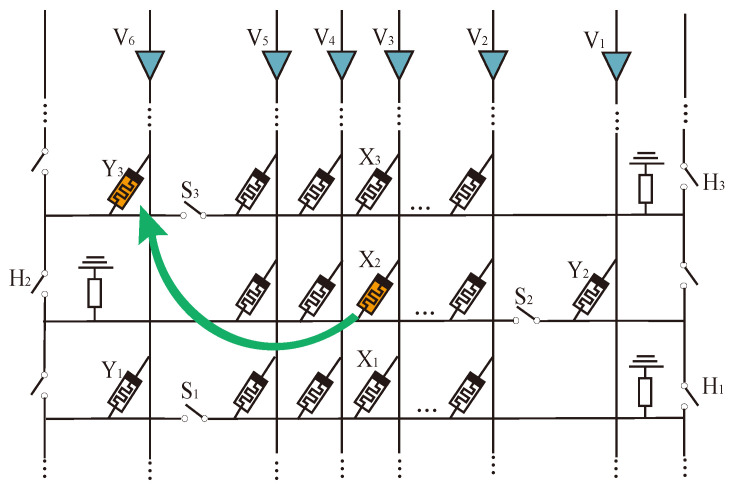
IMPLY operation between different rows in alternating crossbar array.

An alternating crossbar array structure is proposed to realize the rapid data interaction between different rows. As shown in Figure 8, two main differences exist between the alternating crossbar array and the traditional structure. First, the memristors in each column are placed alternately to avoid interference from memristors in adjacent rows in the same column. Second, a column of switches, *H*, is added to isolate the interference of memristors in the back part of the same row. Controlling the switches *H* and *S* allows us to realize fast logic operations in different rows and columns.

In Figure 8, an IMPLY operation is performed on $X_{2}$ and $Y_{3}$. $X_{2}$ is in row 2, column $V_{3}$, and $Y_{3}$ is in row 3, column $V_{6}$. We can perform the following operations at the same time:

(1) Turn on switch $H_{2}$ and turn off all other *H* switches. Select the second and third lines.

(2) Turn off switches $S_{2}$ and $S_{3}$. The second line is connected to the third line and will not affect other lines.

(3) Apply voltage $V_{SET}$ to column $V_{6}$ and voltage $V_{COND}$ to column $V_{3}$, then perform the IMPLY operation involving only $X_{2}$ and $Y_{3}$.

The memory resistance IMPLY operation between different rows can be completed in one step by alternately crossing the array through the above method. Upon analysis, it has been observed that the alternating crossbar array performs faster while calculating memristors involving different rows compared to the traditional crossbar array. The use of an alternating crossbar array enables the completion of simple multiplication in a single step, thereby greatly enhancing the calculation efficiency.

## 3. Data Transfer within MAT

The data movement method in the MAT is realized by logical operation, and the data in the basic crossbar array structure can only be executed in the same row or column, so the data movement must be carried out, and the two operands must be moved to the same row or column. The existing methods are implemented by a MAGIC NOT operation, and a data movement needs to execute NOT logic at least twice, which has low execution efficiency and wastes space for the requirements of temporary storage cells. By analyzing the above problems, this paper proposes an efficient data transfer method, which reserves an auxiliary row and line at the edge of the crossbar array and transfers data with AND and OA logic. The details are as follows:

### 3.1. Structure of mMPU

**Figure 9:**
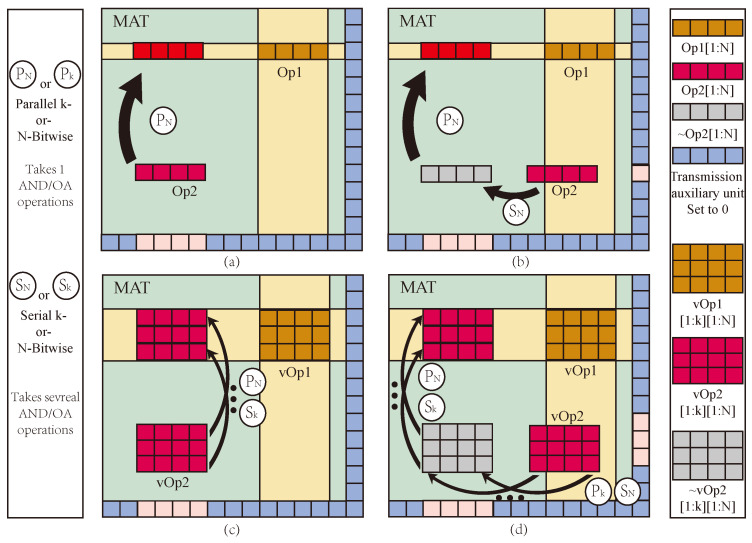
Proposed array structure and data transfer. (**a**) Op1 shares data lines with Op2, (**b**) Op1 shares part data lines with Op2, (**c**) vOp1 shares data lines with vOp2, and (**d**) vOp1 shares part data lines with vOp2.

We adopt the method of adding data transfer auxiliary cells in the array, as shown in Figure 9. To enable both row and column logic operations in the crossbar array, we allocate additional rows and columns at the bottom and right of the array. These cells are set to logical 0 and serve as operands for AND and OR; the destination positions are set to 1. All other cells in the array maintain their original state.

When two vector operands do not overlap, the element in vector Op2 can be moved with AND or OA logic to the destination position parallel with one step. When two operands overlap, the elements in the Op2 vector need to be serially moved to a temporary cell that does not overlap with Op1 and then moved to the destination position.

### 3.2. Design of Data Movement Method

This paper uses AND or OA logic based on IMPLY to realize data transmission. Assuming that the calculation needs to be performed between two operands, Op1 and Op2, in the same MAT, there are two possible situations: the first is that Op2 and Op1 do not share bit lines, as shown in Figure 9a,c, and the other is that the bit lines of Op2 and Op1 partially overlap, as shown in Figure 9b,d.

For the first case, set the destination position data to 1, set the transmission auxiliary cell value (Aux) to 0, and perform OA logic; the input is Op2 and Aux, and the output is destination position. For example, if the data in the I-th row and the j-th column in the array is moved to the K-th row and J-th column cell, then the voltage $V_{COND}^{\prime }$ is applied to the I-th row word line. The voltage $V_{COND}^{\prime }$ is applied to the transmission auxiliary cells. The voltage $V_{CLEAR}$ is applied to the J-th row word line, and the data transfer can be completed according to the basic principle of OA logic. Among them, as shown in Figure 9a, the four data in Op2 are on the same line and can be moved to the destination synchronously. When the operand has multiple vectors, then the vectors can be moved serially, as shown in Figure 9c.

In the second case, because there are some overlapping data between Op1 and Op2, it is necessary to move the data to the location where there is no shared bus by AND or OA logic, as shown in Figure 9b,d. Then, as in the first case, the data transfer can be completed by applying corresponding voltages to the destination location, the transmission data, and the transmission auxiliary cell, respectively, by using OA logic. If there are multiple data groups, it is necessary to transfer the data separately. In the vertical direction, first sequentially move the column data in units to a temporary position, and then horizontally move it in units of row data to the destination position, as shown in Figure 9d. This situation is similar to the MAGIC method, but we use OA logic operation to transfer data, which has more latency and power consumption advantages.

**Figure 10:**
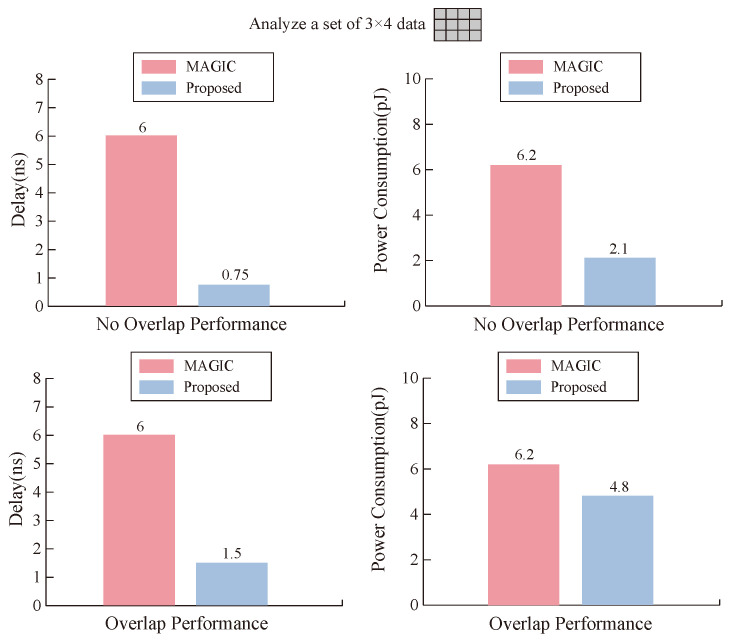
Comparison between our proposed design and MAGIC design in terms of time delay and power consumption.

As seen from Figure 10, when our memristor executes OA and AND logic, it has lower time delay and power consumption than the MAGIC operation. The reason for this result is that $R_{ON}$, $R_{OFF}$, $V_{ON}$, and $R_{OFF}$ of our memristor have smaller values to meet MIMO logic; on the other hand, it also reflects the efficiency of our designed transfer mode.

## 4. Mul-Bit Multiplier Design

### 4.1. Multiplier Principle

**Figure 11:**
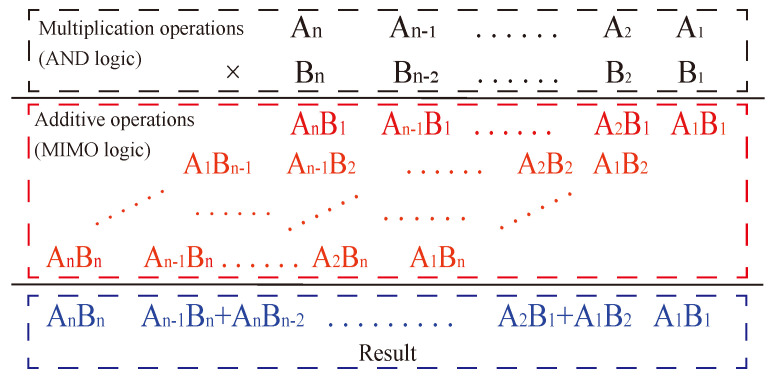
Multiplication formula.

The multiplication formula consists of two parts, as shown in Figure 11, starting with the multiplication operation of multiplying two one-digit numbers, also known as AND logic, followed by the summation of the corresponding position value, also known as an addition. Together, the two constitute the multiplication operation. Therefore, a multiplier can be realized by converting multiplication to addition with multiple digits.

The multiplier consists of multiple full adders and uses a crossbar array structure to convert the multiplication operation into a parallel operation of multiple full adders, which simplifies the operation while optimizing the number of MOS tubes in the circuit, improving the integration of the integrated circuit and making the circuit denser.

**Figure 12:**
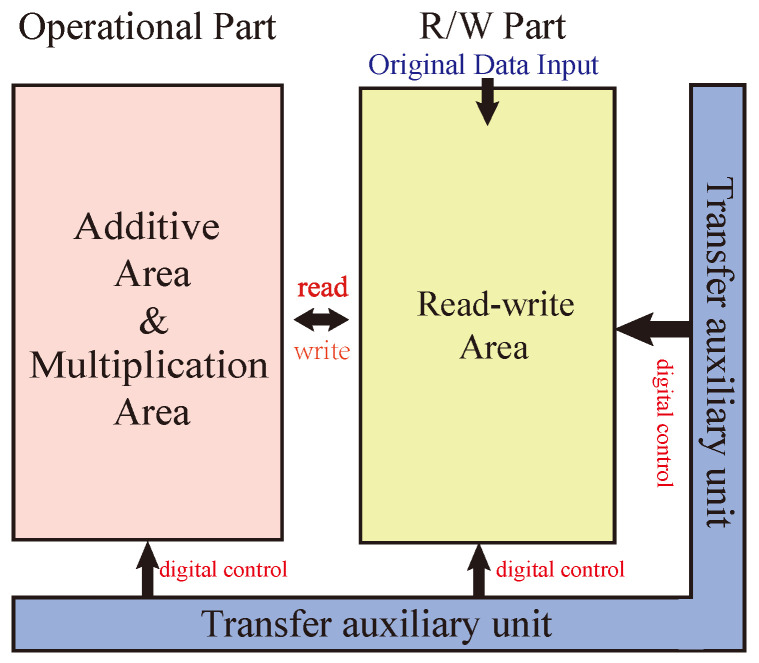
Multiplier structure.

The whole multiplier circuit is divided into three parts: one for arithmetic, one for storage, and one for data transfer auxiliary cells. The structure of the Operational Part adopts the alternating crossbar array structure mentioned previously. In each row, there are several memristors, $A_{i}$, $B_{i}$, …, $X_{i}$, and $M_{i}$, responsible for performing the arithmetic. The memristor $C_{i}$ is responsible for holding the rounding function; the two types of memristors are separated by switches, $S_{i}$. Between the rows, there are cross-arranged switches, $H_{i}$, for the control of the steps of the operations. The Operational Part is required to perform both multiplication and addition calculations. The read/write section (R/W Part) is responsible for writing and reading data, and this section can write and save the data from being calculated or the data output from the Operational Part, or it can read the data from this section and input it to the Operational Part for calculation. The data transmission auxiliary unit is a series of memristors located at the right and bottom of the array and is used to assist the transfer of data, which is usually set to logic 0 to facilitate the execution of OA logic. All these three sections form the multiplier’s arithmetic circuit, as shown in Figure 12.

### 4.2. Multiplier Operations

**Figure 13:**
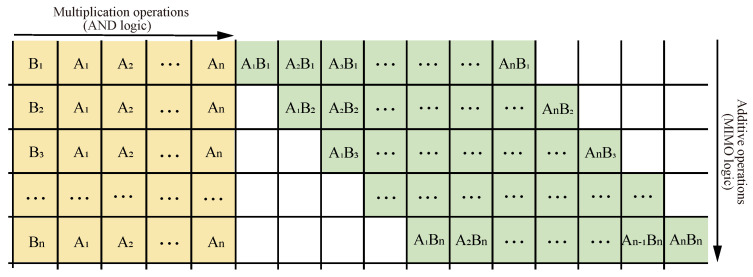
Multiplier circuit data layout.

**Figure 14:**
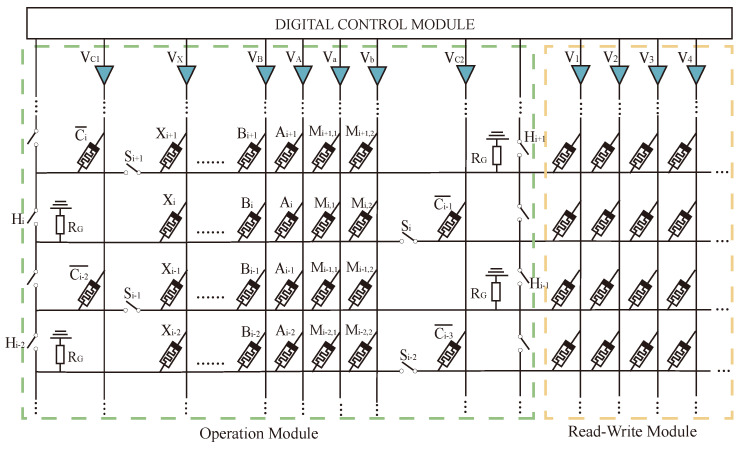
Multiplier simulation circuit (Take a 2×2 multiplier as an example).

Take a 2×2 multiplier as an example; the multipliers are $A_{1}$, $A_{2}$ and $B_{1}$, $B_{2}$. The operation steps of this multiplier are as follows:

Step 1: Read and input the data from the read/write area into the multiplication area of the operational part through the designed transmission method. The layout of the multiplication area is shown in the yellow part in Figure 13. For a 2×2 multiplier, $B_{1}$ stores in memristor $A_{i+3}$, $B_{2}$ stores in memristor $B_{i+3}$, $A_{1}$ stores in memristor $A_{i+2}$ and $B_{i+2}$, and $A_{2}$ stores in memristor $A_{i+1}$ and $B_{i+1}$, as shown in Figure 14.

Step 2: Perform the multiplication operation. The logic of the operation is AND logic, and the truth table of the logic is shown in Table 3; the multiplication obtains four results: $A_{1}$$B_{1}$, $A_{1}$$B_{2}$, $A_{2}$$B_{1}$, and $A_{2}$$B_{2}$. The result data will be directly input into the additive area. As shown in the green part in Figure 13, $A_{1}$$B_{1}$ stores in memristor $A_{i}$, $A_{1}$$B_{2}$ stores in memristor $B_{i-1}$, $A_{2}$$B_{1}$ stores in memristor $A_{i-1}$, and $A_{2}$$B_{2}$ stores in memristor $A_{i-2}$. For a 2×2 multiplier, the addition area will be performed on three rows in the next few steps. $A_{1}$$B_{1}$ and 0 are added together, $A_{1}$$B_{2}$ and $A_{2}$$B_{1}$ are added together, and $A_{2}$$B_{2}$ and 0 are added together.

Steps 3 and 4: Turn on the switches $S_{i-2}$, $S_{i-1}$, and $S_{i}$. Disconnect the switches on all columns. The full adder module can calculate step 3 and step 4 simultaneously. At this stage, the multiplier only needs two steps through parallel calculation.

Steps 5 and 6: Turn on the switches $H_{i-2}$ and $H_{i-1}$, $H_{i}$. Disconnect the switch $S_{i-2}$, $S_{i-1}$, $S_{i}$, and all other switches. In this way, the memristor $\bar{C_{i}}$ can be connected to $L_{i}$, the memristor $\bar{C_{i-1}}$ can be connected to $L_{i-1}$, and the memristor $\bar{C_{i-2}}$ can be connected to $L_{i-2}$ without affecting other bits, so data transmission can be realized between the carrier and the standard. As in steps 3 and 4, the multiplier can also complete steps 5 and 6 in parallel. At this stage, parallel computing requires two steps.

Step 7: Turn on the switches $S_{i-2}$, $S_{i-1}$, $S_{i}$ and $H_{i-2}$, $H_{i-1}$, $H_{i}$. Disconnect all other switches. By preparing steps 3 to 6 and applying an alternating crossbar array, we can complete the current bit’s carry calculation in one step.

Step 8 to Step 12: Turn on the switch $S_{i-2}$, $S_{i-1}$, $S_{i}$. Disconnect the switches on all columns. After completing step 7, $L_{i+1}$, $L_{i}$, $L_{i-1}$ receive the carry from the adjacent lower bits, and each bit can independently complete all the remaining steps. Therefore, the full adder module can complete the next steps in parallel. At this stage, the parallel calculation of the multiplier takes five steps. All the operation steps are shown in Table 4. There are a total of 12 steps in a 2×2 multiplication operation.

Next, we can extend the 2×2 multiplier to an n×n multiplier with the same steps as the 2×2 multiplier; the first four steps are the same as those of the 2×2 multiplier, while in the steps of the addition algorithm, for multi-bit addition, we need to add every two values of the additive number and store the intermediate value into the read–write region. Thus, the steps of the addition algorithm are (n − 1)(n + 9). The n×n bit multiplier requires total steps $1+\left(n-1\right)\left(n+9\right)=n^{2}+8n-8$ steps.

## 5. Simulation and Analysis

### 5.1. Simulation

The proposed data transfer and multiplier are simulated with PSpice(v17.2) simulation tools to verify their correctness. The VTEAM model is selected as the memristor model. The simulated circuit is shown in Figure 14, and the memristor model parameters are shown in Table 5. The power consumption and time delay of some logic operations used in the operation under the given memristor parameters are shown in Table 6.

Taking the 2×2 multiplier operation as an example, all data transmission modes of this circuit are the transfer-line method mentioned above. In the circuit, firstly, the values to be calculated, $A_{1}$, $A_{2}$, $B_{1}$, and $B_{2}$, are deposited into the read–write area on the right side. After that, the data are transferred to the multiplication module through the digital control module to carry out the AND operation, $A_{1}$ × $B_{1}$, $A_{1}$ × $B_{2}$, $A_{2}$ × $B_{1}$, $A_{2}$ × $B_{2}$, and the results, $A_{1}$$B_{1}$, $A_{1}$$B_{2}$, $A_{2}$$B_{1}$, and $A_{2}$$B_{2}$ are returned to the read–write after the operation is finished, clearing the multiplication region to participate in the next addition operation. Then, the four data are input into the corresponding memristors according to Figure 13; memristor $B_{i}$ stores $A_{2}$$B_{2}$, memristor $B_{i-1}$ stores $A_{2}$$B_{1}$, memristor $A_{i-1}$ stores $A_{1}$ × $B_{2}$, memristor $A_{i-2}$ stores $A_{1}$ × $B_{1}$, and memristor $\bar{C_{i}}$ stores the rounding data. The final multiplication result is obtained by performing a full adder operation.

Regarding power consumption, the logic operation power consumption required for each step of the multiplier is shown in Table 6. The advantage of a staggered cross array is that it can convert two rows into one row and complete the carry in one step. Take a 32-bit multiplier as an example; it needs a total of 992 carries at most, and each carry is completed in one step, with a power consumption of 0.227 pJ and a total power consumption of 225.184 pJ. If we use the same device to calculate in the traditional cross array, we can see from Figure 7 that it takes at least two steps to complete a carry. The power consumption of peer movement is 0.235 pJ, and the power consumption of the carry is 0.227 pJ, so the power consumption of one step bit operation is the sum of the above two, and the total power consumption required for the carry is 458.304 pJ. With the increase of computing bits, the power consumption of the staggered array decreases more significantly.

**Figure 15:**
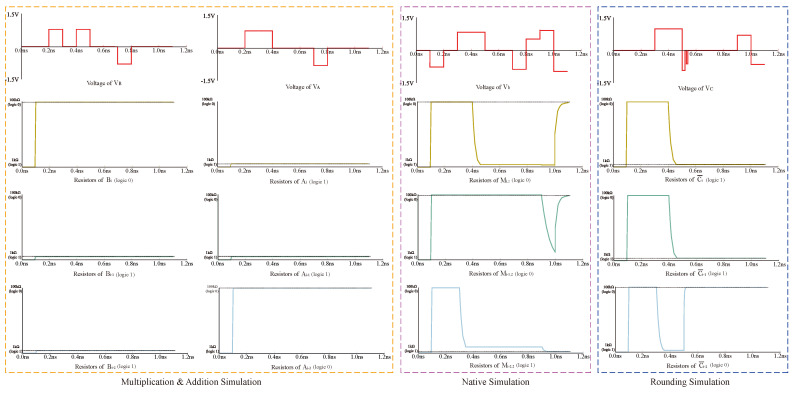
2 × 2 Multiplication result 11 × 11 = 1001 ($\bar{C_{i}}$, $M_{i,2}$, $M_{i-1,2}$, $M_{i-2,2}$).

We input the multiplier for 11 and 11; the multiplication result should be 1001. This simulation mainly verifies steps 3 to 12, and the first few steps are relatively simple, so the simulation is not carried out. The simulation results as shown in Figure 15; 0 state stands for the state of $R_{off}$, the resistance of 100 kΩ, and 1 state stands for $R_{on}$, the resistance of 1 kΩ. The four resistors in the figure, $\bar{C_{i}}$, $M_{i,2}$, $M_{i-1,2}$, $M_{i-2,2}$, respectively, correspond to the result of the four bits. The resistance values of the four memristors finally reach stability after 1ns, and the result is 1001, which is in line with the multiplication logic, and the simulation is successful.

**Figure 16:**
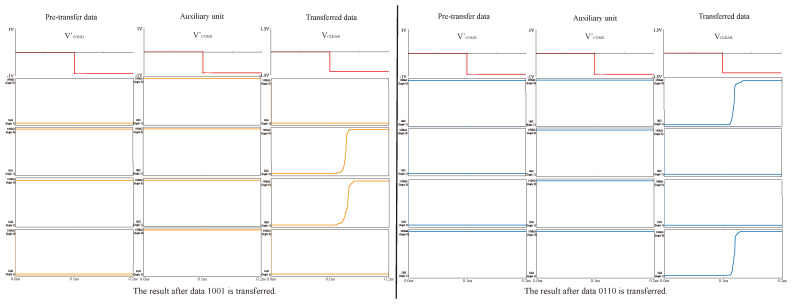
Data transfer simulation.

In addition, we also simulated the reliability of data transmission. We simulated the data transmission of data 1001 and another group of multiplication results, 0110, and the results are shown in Figure 16. By applying voltage $V_{COND}^{\prime }$ to the pre-transfer data and voltage $V_{CLEAR}$ to the transferred data, we can see that the data in the register area has changed from logic 1 to corresponding data to be saved. These prove the success of data transfer.

### 5.2. Analysis and Comparison

From the simulated circuit and the multiplier operation steps, we can conclude that the number of memristors used in our n-bit multiplier is $n^{2}+2n+n^{2}+n=2n^{2}+3n$, and the number of switches used is 2n+2n=4n. As can be seen from Table 7, comparing our multiplier with other multipliers, our design has a significant advantage in multiplication steps and the number of switches over Shift & Add type multipliers. Compared to the crossbar array multiplier, our design performs better regarding the number of memristors and switches. Compared to the SEMI-SERIAL multiplier, our design performs better regarding the number of steps and switches, and there is little difference in the number of memristors.

**Figure 17:**
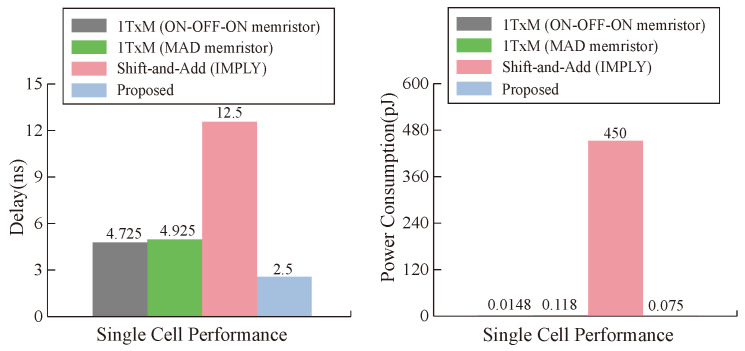
Delay and energy comparison of a single cell.

**Figure 18:**
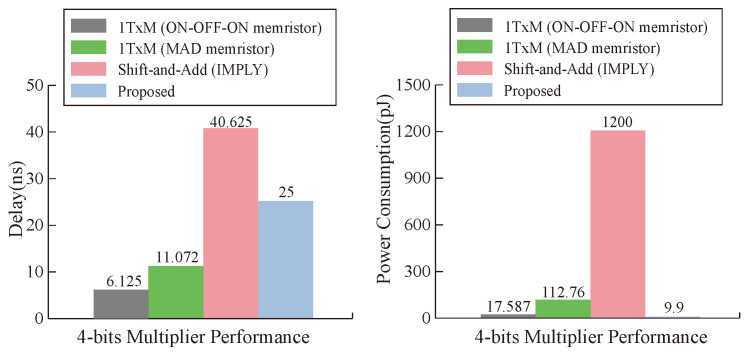
Delay and energy comparisons between multiplication approaches.

From the point of view of a single operation cell, our proposed multiplier is superior to other multipliers in terms of latency and power consumption under the condition of ignoring the latency of data transmission, and the maximum performance improvement of latency and power consumption reaches 80% and 99%, respectively, as can be seen in Figure 17. From the analysis of the whole multiplier module, our memristor is ahead of the three types of multipliers in power consumption. In terms of latency, the latency of our multiplier is close to that of the 1TxM multiplier [50], but it is obviously improved compared with the shift-and-add multiplier [51], as can be seen in Figure 18. Our work has obvious advantages when compared with other work results, which is due to the parameter selection of our memristor and the efficiency of our designed multiplier.

**Table 7:** Comparison of multipliers for n = 32.

Design	Number of Memristors			Number of Steps			Number of Switches		
	**Total**	**n = 32**	**Imp.**	**Total**	**n = 32**	**Imp.**	**Total**	**n = 32**	**Imp.**
Shift & Add [51]	7n + 1	225	−89%	2n^2^+21n	2720	53%	8n−1	255	50%
Array [47]	7n^2^ − 8n + 9	6921	69%	24n − 35	733	−73%	8n^2^ − 8n + 9	7945	98%
SEMI-SERIAL [48]	2n^2^ + n + 2	2082	−3%	[$log_{2}$n](10n + 2) + 4n + 2	1740	26%	12[n/2] + [(n − 1)/2]	207	38%
Proposed	2n^2^+3n	2144	-	n^2^ + 8n − 8	1272	-	4n	128	-

## 6. Conclusions

This paper proposes an efficient PiM MAT data transmission method based on the alternating crossbar array and further proposes a multi-bit multiplier design scheme. The proposed data transmission method reserves the row and column edges as temporary assistant cells, employs OA logic as the moving logic, significantly reduces data transmission steps, and improves efficiency. The proposed multi-bit multiplier converts multiplication operations to multi-bit addition operations, greatly improving multiplier execution efficiency. Compared to existing memristive multipliers, it has lower latency and power consumption, higher reliability, and greater flexibility. This work is of great significance for promoting research on high-performance PiM.

Our model is based on the ideal memristor, and the control voltage needs to be adjusted according to the actual memristor characteristics when it is implemented. In addition, the problem of leakage current is not discussed in this paper. In the following research, it is necessary to comprehensively evaluate the characteristics of power consumption, delay, storage density, and so on, according to the specific implementation process to provide guidance for circuit design.

As the number of calculation bits increases, the proposed multiplier’s latency gradually increases, which is not conducive to its fast and efficient operation. In the future, we will simplify the multiplier’s operation steps to gradually improve the problem of excessive time extension.

## Figures and Tables

**Table 1:** ONO logical truth table.

	P1	P2	Q	Result
case 1	0	0	q	1
case 2	0	1	q	q
case 3	1	0	q	q
case 4	1	1	q	q

**Table 2:** OA logical truth table.

	P1	P2	Q	Result
case 1	0	0	q	0
case 2	0	1	q	q
case 3	1	0	q	q
case 4	1	1	q	q

**Table 3:** AND logical truth table.

	P	Q	Result
case 1	0	0	0
case 2	0	1	0
case 3	1	0	0
case 4	1	1	1

**Table 4:** Implementation of the 2×2 multiplier.

Step	Operation	Voltage	The Logical Value after Operation in:		
			$M_{i,1}$	$M_{i,2}$	$\bar{C_{i}}$
1	Read and Input the Data (R/W Part to Multiplier area of Operational Part)	Digital Control	0	0	0
2	The multiplication area performs AND logic operation.	Digital Control	0	0	0
3	The addition region performs addition operation/$CLEAR\;(M_{i,1},\;M_{i,2},\;\bar{C_{i}})$	$V_{C1}=V_{a}=V_{b}=V_{C2}=V_{CLEAR}$	0	0	0
4	$\left(A_{i},\;B_{i}\right)\;ONO\;M_{i,1}$	$V_{B}=V_{A}=V_{COND};\;V_{a}=V_{SET}$	$\bar{A_{i}+B_{i}}$	0	0
5	$B_{i}\;IMPLY\;\left(M_{i,2},\;\bar{C_{i}}\right)$	$V_{A}=V_{COND};\;V_{C1}=V_{b}=V_{C2}=V_{SET}$	$\bar{A_{i}+B_{i}}$	$\bar{B_{i}}$	$\bar{B_{i}}$
6	$A_{i}\;IMPLY\;\left(M_{i,2},\;\bar{C_{i}}\right)$	$V_{B}=V_{COND};\;V_{C1}=V_{b}=V_{C2}=V_{SET}$	$\bar{A_{i}+B_{i}}$	$\bar{A_{i}}+\bar{B_{i}}$	$\bar{A_{i}}+\bar{B_{i}}$
7	$\left(\bar{C_{i-1}},\;M_{i,1}\right)\;OA\;\bar{C_{i}}$	$\left\{\begin{array}{cc} V_{C1}=V_{a}=V_{COND}^{\prime };\;V_{C2}=V_{CLEAR} & i\in odd\\ V_{a}=V_{C2}=V_{COND}^{\prime };\;V_{C1}=V_{CLEAR} & i\in even \end{array}\right.$	$\bar{A_{i}+B_{i}}$	$\bar{A_{i}}+\bar{B_{i}}$	$\bar{C_{i}}$ (carry-out)
8	$CLEAR\;M_{i,1}$	$V_{a}=V_{CLEAR}$	0	$\bar{A_{i}}+\bar{B_{i}}$	-
9	$\left(A_{i},\;B_{i}\right)\;OA\;M_{i,2}$	$V_{B}=V_{A}=V_{COND}^{\prime };\;V_{b}=V_{CLEAR}$	0	$A_{i}⊕B_{i}$	-
10	$M_{i,2}\;IMPLY\;M_{i,1}$	$V_{b}=V_{COND};\;V_{a}=V_{SET}$	$\bar{A_{i}⊕B_{i}}$	$A_{i}⊕B_{i}$	-
11	$\bar{C_{i-1}}\;IMPLY\;M_{i,2}$	$V_{C1}=V_{C2}=V_{COND};\;V_{b}=V_{SET}$	$\bar{A_{i}⊕B_{i}}$	$C_{i-1}+A_{i}⊕B_{i}$	-
12	$\left(\bar{C_{i-1}},\;M_{i,1}\right)\;OA\;M_{i,2}$	$V_{C1}=V_{a}=V_{C2}=V_{COND}^{\prime };\;V_{b}=V_{CLEAR}$	$\bar{A_{i}⊕B_{i}}$	$S_{i}$ (sum)	-

The first step and the second step are only carried out once in the whole operation process, and the remaining steps are carried out simultaneously in the order of each line in the table.

**Table 5:** Memristors and circuit parameters considered in the simulations.

*a*	*p*	$k_{\mathrm{on}}$	$k_{\mathrm{off}}$	$V_{\mathrm{CLOSE}}$	$V_{\mathrm{OPEN}}$	$R_{\mathrm{ON}}$	$R_{\mathrm{OFF}}$	$V_{\mathrm{SET}}$	$V_{\mathrm{COND}}$	$V_{\mathrm{CLEAR}}$	$V_{\mathrm{COND}}^{\prime }$	$R_{G}$
200	200	1250	1250	1 V	−1 V	1 kΩ	100 kΩ	1.2 V	0.8 V	−1.2 V	−0.8 V	500 Ω

**Table 6:** The power consumption and time delay of some logic operations.

	Time Delay (ns)	Power Consumption (pJ)
Single Memristor	0.25	0.075
OA	0.31	0.227
AND	0.271	0.161
MIMO IMPLY	0.263	0.235
ONO	0.28	0.229

## Data Availability

The datasets generated during and/or analyzed during the current study are available from the corresponding author on reasonable request.

## References

[1] Ahn J., Hong S., Yoo S., Mutlu O., Choi K. A scalable processing-in-memory accelerator for parallel graph processing. Proceedings of the 42nd Annual International Symposium on Computer Architecture.

[2] Hur R.B., Kvatinsky S. Memory processing cell for in-memory processing. Proceedings of the 2016 IEEE/ACM International Symposium on Nanoscale Architectures (NANOARCH).

[3] Chua L. (1971). Memristor-the missing circuit element. IEEE Trans. Circuit Theory.

[4] Strukov D.B., Snider G.S., Stewart D.R., Williams R.S. (2008). The missing memristor found. Nature.

[5] Xiao Y., Jiang B., Zhang Z., Ke S., Jin Y., Wen X., Ye C. (2023). A review of memristor: Material and structure design, device performance, applications and prospects. Sci. Technol. Adv. Mater..

[6] Kvatinsky S., Ramadan M., Friedman E.G., Kolodny A. (2015). VTEAM: A General Model for Voltage-Controlled Memristors. IEEE Trans. Circuits Syst. II Express Briefs.

[7] Deng Q., Wang C., Sun J., Sun Y., Jiang J., Lin H., Deng Z. (2024). Nonvolatile CMOS Memristor, Reconfigurable Array, and Its Application in Power Load Forecasting. IEEE Trans. Ind. Inform..

[8] Wu H., Bao B., Liu Z., Xu Q., Jiang P. (2016). Chaotic and periodic bursting phenomena in a memristive Wien-bridge oscillator. Nonlinear Dyn..

[9] Li X., Sun J., Ma W., Sun Y., Wang C., Zhang J. (2024). Adaptive biomimetic neuronal circuit system based on Myelin sheath function. IEEE Trans. Consum. Electron..

[10] Wan Q., Liu J., Liu T., Sun K., Qin P. (2024). Memristor-based circuit design of episodic memory neural network and its application in hurricane category prediction. Neural Netw..

[11] Lin H., Wang C., Sun J., Zhang X., Sun Y., Iu H.H.C. (2024). Memristor-coupled asymmetric neural networks: Bionic modeling, chaotic dynamics analysis and encryption application. Chaos Solitons Fractals.

[12] Li X., Sun J., Sun Y., Wang C., Hong Q., Du S., Zhang J. (2023). Design of Artificial Neurons of Memristive Neuromorphic Networks Based on Biological Neural Dynamics and Structures. IEEE Trans. Circuits Syst. Regul. Pap..

[13] Yao W., Liu J., Sun Y., Zhang J., Yu F., Cui L., Lin H. (2024). Dynamics analysis and image encryption application of Hopfield neural network with a novel multistable and highly tunable memristor. Nonlinear Dyn..

[14] Yuan R., Pek Jun T., Lei C., Yang Z., Liu C., Zhang T., Ge C., Huang R., Yang Y. (2023). A neuromorphic physiological signal processing system based on VO2 memristor for next-generation human-machine interface. Nat. Commun..

[15] Peng L., Can L., Zhongrui W., Yunning L., Hao J., Wenhao S., Mingyi R., Ye Z., Upadhyay N.K., Barnell M. (2020). Three-dimensional memristor circuits as complex neural networks. Nat. Electron..

[16] Tang D., Wang C., Lin H., Yu F. (2024). Dynamics analysis and hardware implementation of multi-scroll hyperchaotic hidden attractors based on locally active memristive Hopfield neural network. Nonlinear Dyn..

[17] Bao B., Hu J., Cai J., Zhang X., Bao H. (2023). Memristor-induced mode transitions and extreme multistability in a map-based neuron model. Nonlinear Dyn..

[18] Zhang S., Li C., Zheng J., Wang X., Zeng Z., Chen G. (2021). Generating Any Number of Diversified Hidden Attractors via Memristor Coupling. IEEE Trans. Circuits Syst. Regul. Pap..

[19] Ma M., Lu Y. (2024). Synchronization in scale-free neural networks under electromagnetic radiation. Chaos Interdiscip. J. Nonlinear Sci..

[20] Lehtonen E., Poikonen J.H., Laiho M., Kanerva P. (2014). Large-Scale Memristive Associative Memories. IEEE Trans. Very Large Scale Integr. (VLSI) Syst..

[21] Wang X., Li S., Liu H., Zeng Z. (2018). A Compact Scheme of Reading and Writing for Memristor-Based Multivalued Memory. IEEE Trans.-Comput.-Aided Des. Integr. Circuits Syst..

[22] Sun J., Jiang M., Zhou Q., Wang C., Sun Y. (2022). Memristive cluster based compact high-density nonvolatile memory design and application for image storage. Micromachines.

[23] Sun J., Kang K., Sun Y., Hong Q., Wang C. (2022). A multi-value 3D crossbar array nonvolatile memory based on pure memristors. Eur. Phys. J. Spec. Top..

[24] Zangeneh M., Joshi A. (2014). Design and Optimization of Nonvolatile Multibit 1T1R Resistive RAM. IEEE Trans. Very Large Scale Integr. (VLSI) Syst..

[25] Sun J., Li M., Kang K., Zhu S., Sun Y. (2018). Design of heterogeneous memristor based 1T2M multi-value memory crossbar array. J. Electron. Inf. Technol..

[26] Teimoory M., Amirsoleimani A., Ahmadi A., Ahmadi M. (2018). A 2M1M Crossbar Architecture: Memory. IEEE Trans. Very Large Scale Integr. (VLSI) Syst..

[27] Im I.H., Kim S.J., Jang H.W. (2020). Memristive devices for new computing paradigms. Adv. Intell. Syst..

[28] Yao P., Wu H., Gao B., Tang J., Zhang Q., Zhang W., Yang J.J., Qian H. (2020). Fully hardware-implemented memristor convolutional neural network. Nature.

[29] Chi P., Li S., Xu C., Zhang T., Zhao J., Liu Y., Wang Y., Xie Y. (2016). Prime: A novel processing-in-memory architecture for neural network computation in reram-based main memory. ACM Sigarch Comput. Archit. News.

[30] Yao P., Wu H., Gao B., Eryilmaz S.B., Huang X., Zhang W., Zhang Q., Zhang Q., Deng N., Shi L. (2017). Face classification using electronic synapses. Nat. Commun..

[31] Ben Hur R., Kvatinsky S. Memristive memory processing cell (MPU) controller for in-memory processing. Proceedings of the 2016 IEEE International Conference on the Science of Electrical Engineering (ICSEE).

[32] Talati N., Ali A.H., Hur R.B., Wald N., Ronen R., Gaillardon P., Kvatinsky S. Practical challenges in delivering the promises of real processing-in-memory machines. Proceedings of the Design, Automation & Test in Europe Conference & Exhibition (DATE).

[33] Sun Z., Ambrosi E., Bricalli A., Ielmini D. (2018). Logic computing with stateful neural networks of resistive switches. Adv. Mater..

[34] Singh T. (2015). Hybrid memristor-cmos (memos) based logic gates and adder circuits. arXiv.

[35] Liu G., Shen S., Jin P., Wang G., Liang Y. (2021). Design of memristor-based combinational logic circuits. Circuits Syst. Signal Process..

[36] Borghetti J., Snider G.S., Kuekes, Yang J.J., Stewart D.R., Williams R.S. (2010). ‘Memristive’switches enable ‘stateful’logic operations via material implication. Nature.

[37] Kvatinsky S., Belousov D., Liman S., Satat G., Wald N., Friedman E.G., Kolodny A., Weiser U.C. (2014). MAGIC—Memristor-aided logic. IEEE Trans. Circuits Syst. II Express Briefs.

[38] Jiang M., Sun J., Wang C., Liao Z., Sun Y., Hong Q., Zhang J. (2023). An efficient memristive alternating crossbar array and the design of full adder. Nonlinear Dyn..

[39] Kvatinsky S., Satat G., Wald N., Friedman E.G., Kolodny A., Weiser U.C. (2023). Memristor-based material implication (IMPLY) logic: Design principles and methodologies. IEEE Trans. Very Large Scale Integr. (VLSI) Syst..

[40] Rohani S.G., TaheriNejad N. An improved algorithm for IMPLY logic based memristive full-adder. Proceedings of the 2017 IEEE 30th Canadian Conference on Electrical and Computer Engineering (CCECE).

[41] Rohani S.G., Taherinejad N., Radakovits D. (2020). A Semiparallel Full-Adder in IMPLY Logic. IEEE Trans. Very Large Scale Integr. (VLSI) Syst..

[42] Sun J., Peng M., Jiang H., Hong Q., Sun Y. (2022). HMIAN: A hierarchical mapping and interactive attention data fusion network for traffic forecasting. IEEE Internet Things J..

[43] Amirsoleimani A., Alibart F., Yon V., Xu J., Pazhouhandeh M.R., Ecoffey S., Beilliard Y., Genov R., Drouin D. (2020). In-Memory Vector-Matrix Multiplication in Monolithic Complementary Metal–Oxide–Semiconductor-Memristor Integrated Circuits: Design Choices, Challenges, and Perspectives. Adv. Intell. Syst..

[44] Haghiri S., Nemati A., Feizi S., Amirsoleimani A., Ahmadi A., Ahmadi M. A memristor based binary multiplier. Proceedings of the 2017 IEEE 30th Canadian Conference on Electrical and Computer Engineering (CCECE).

[45] Guckert L., Swartzlander E.E. Dadda Multiplier designs using memristors. Proceedings of the 2017 IEEE International Conference on IC Design and Technology (ICICDT).

[46] Teimoory M., Amirsoleimani A., Ahmadi A., Ahmadi M. (2018). A hybrid memristor-CMOS multiplier design based on memristive universal logic gates. Int. Midwest Symp. Circuits Syst..

[47] Yu S., Shafik R., Bunnam T., Chen K., Yakovlev A. Self-Amplifying Current-Mode Multiplier Design using a Multi-Memristor Crossbar Cell Structure. Proceedings of the 2020 27th IEEE International Conference on Electronics, Circuits and Systems (ICECS).

[48] Radakovits D., TaheriNejad N., Cai M., Delaroche T., Mirabbasi S. (2020). A memristive multiplier using semi-serial imply-based adder. IEEE Trans. Circuits Syst. Regul. Pap..

[49] Huang P., Kang J., Zhao Y., Chen S., Han R., Zhou Z., Chen Z., Ma W., Li M., Liu L. (2016). Reconfigurable nonvolatile logic operations in resistance switching crossbar array for large-scale circuits. Adv. Mater..

[50] Yu S., Shafik R., Bunnam T., Chen K., Yakovlev A. Optimized multi-memristor model based low energy and resilient current-mode multiplier design. Proceedings of the 2021 Design, Automation & Test in Europe Conference & Exhibition (DATE).

[51] Guckert L., Swartzlander E.E. (2017). Optimized memristor-based multipliers. IEEE Trans. Circuits Syst. Regul. Pap..

